# Cytoplasmic and/or Nuclear Expression of β-Catenin Correlate with Poor Prognosis and Unfavorable Clinicopathological Factors in Hepatocellular Carcinoma: A Meta-Analysis

**DOI:** 10.1371/journal.pone.0111885

**Published:** 2014-11-17

**Authors:** Jiang Chen, Jinghua Liu, Renan Jin, Jiliang Shen, Yuelong Liang, Rui Ma, Hui Lin, Xiao Liang, Hong Yu, Xiujun Cai

**Affiliations:** 1 Department of General Surgery, Sir Run Run Shaw Hospital of Zhejiang University, Hangzhou, Zhejiang, China; 2 Department of Surgery, Zhejiang University Hospital, Hangzhou, Zhejiang, China; Heinrich-Heine-University and University Hospital Duesseldorf, Germany

## Abstract

**Background:**

The β-catenin is an important effector in WNT/β-catenin signaling pathway, which exerts a crucial role in the development and progression of hepatocellular carcinoma (HCC). Some researchers have suggested that the overexpression of β-catenin in cytoplasm and/or nucleus was closely correlated to metastasis, poor differentiation and malignant phenotype of HCC while some other researchers hold opposite point. So far, no consensus was obtained on the prognostic and clinicopathological significance of cytoplasmic/nuclear β-catenin overexpression for HCCs.

**Methods:**

Systematic strategies were applied to search eligible studies in all available databases. Subgroup analyses, sensitivity analyses and multivariate analysis were performed. In this meta-analysis, we utilized either fixed- or random-effects model to calculate the pooled odds ratios (OR) and its 95% confidence intervals (CI).

**Results:**

A total of 22 studies containing 2334 cases were enrolled in this meta-analysis. Pooled data suggested that accumulation of β-catenin in cytoplasm and/or nucleus significantly correlated with poor 1-, 3- and 5-year OS and RFS. Moreover, nuclear accumulation combined with cytoplasmic accumulation of β-catenin tended to be associated with dismal metastasis and vascular invasion while cytoplasmic or nuclear expression alone showed no significant effect. Besides, no significant association was observed between cytoplasmic and/or nuclear β-catenin expression and poor differentiation grade, advanced TNM stage, liver cirrhosis, tumor size, tumor encapsulation, AFP and etiologies. Additional subgroup analysis by origin suggested that the prognostic value and clinicopathological significance of cytoplasmic and/or nuclear β-catenin expression was more validated in Asian population. Multivariate analyses of factors showed that cytoplasmic and/or nuclear β-catenin expression, as well as TNM stage, metastasis and tumor size, was an independent risk factors for OS and RFS.

**Conclusions:**

Cytoplasmic and/or nuclear accumulation of β-catenin, as an independent prognostic factor, significantly associated with poor prognosis and deeper invasion of HCC, and could serve as a valuable prognostic predictor for HCC.

## Introduction

Hepatocellular cancer (HCC) is a global health problem and its incidence has been increasing dramatically since 20 years especially in developed countries [1]. In 2012, it was reported that its annual incidence reached more than half a million worldwide [2]. It ranks No. three on the most frequent cause of cancer-related death list among the global population [3]. Surgery is the main curative treatment, but less than 50% patients survive more than a year following treatment for the poor prognosis of HCC [4]. Until now, few systemic therapies demonstrated a fully positive impact on the prognosis for patients with HCC. Additionally, sorafenib, a multikinase inhibitor, currently used as the targeted anticancer agent, only exhibited comparative efficacy and safety in advanced HCC considering its complicated histologic response and various differentiation of cases [5], [6]. Therefore, it is mandatory to have an elemental understanding of the genes and signaling pathways involved in the initiation and progression of this neoplasm and develop more effective therapies to intervene in this process.

Several molecular pathways are implicated in the hepatic oncogenesis such as β-catenin, p53, EGF, HGF, TGF β and others [7]. The WNT/β-catenin mediated signaling pathway has been well studied and exerted an indispensible role in HCC pathogenesis [8]. The aberrantly activated WNT signaling is usually caused by somatic mutations, which contains several hot-spot mutations present in the *CSF1R, CTNNB1, KRAS, BRAF, NRAS, ERBB2, MET, PIK3CA, JAK1*, and *SMO* genes [9]. Among these hot-spot genes, the mutations of *CTNNB1* gene account for the majority of somatic mutations and appear to be the most common cause for activation of WNT signaling pathway. *CTNNB1* is the coding gene for β-catenin, a multifunctional protein that integrates the intercellular E-cadherin–catenin adhesion system with intracellular WNT signaling pathway.

In normal hepatocytes, the great majority of β-catenin is located in cytomembrane where it directly connects the E-catherin to a-catenin, which is in turn bound to the actin-based cytoskeleton, forming an adhesion complex [10], [11]. And the unbound cytoplasmic β-catenin is kept at a low level by forming a destruction complex with GSK3β, Axin1, Casein Kinase Iα (CKIα) and APC (Adenomatous Polyposis Coli protein) [12]. The cadherin–catenin adhesion complex can regulate cell-cell adhesion and recognition and hence establish and maintain tissue architecture and function. And the destruction complex can be degraded by undergoing phosphorylation and ubiquitination and hence the unbound β-catenin is removed from cytosol, thereby preventing its translocation to the nucleus [12], [13]. When *CTNNB1* mutations occurred, the functional residues of β-catenin may be affected so that the targets usually become invalid of priming phosphorylation by GSK-3β and subsequent catalyzation by proteasome system [14]. Therefore, the unbound β-catenin cannot be removed from the cytosol and accumulates in cytoplasm. The accumulated β-catenin in the cytoplasm could translocate to the nucleus where it serves as a co-factor for the T cell factor (TCF) family of transcription factors to activate the downstream target genes relevant to cell proliferation, migration, invasion, cell cycle progression and metastasis, including *c-myc*, *cyclin-D1*, and *survivin* [15]. Finally, the WNT signaling pathway is activated in the context of *CTNNB1* mutations, though the aberrant activation may also occurred in the absence of *CTNNB1* mutations.

The normal hepatocytes are transformed to the malignant ones when the WNT pathway is initiated. Aberrant activation of WNT/β-catenin signaling prevents the formation of the β-catenin destruction complex and further leads to the accumulation of β-catenin in the cytoplasm and/or nucleus [16]. Thus, the cytoplasmic and/or nuclear expression of β-catenin exhibits close relationship with the activated WNT signaling pathway and thereby the hepatic oncogenesis.

Abnormal cytoplasmic and/or nuclear accumulation of β-catenin has been demonstrated in 17–40% of HCCs [17], indicating that β-catenin may be a potential molecular marker for disease development and progression for patients with HCC. In this regard, vast work has been done to investigate the association of cytoplasmic and/or nuclear β-catenin expression with clinicopathological features, etiologies and prognosis for patients with HCC. However, results about their correlation reported by researchers from different institutions or organizations are highly variable and contradictory. Additionally, the number of cases enrolled in each study was not large enough. Therefore, it is necessary to conduct a systematic and comprehensive analysis to achieve a reasonable consensus about the prognostic and clinicopathological significance of β-catenin expression in cytoplasm and/or nucleus.

In this paper, a meta-analysis was performed based on retrospective studies to evaluate the prognostic value and clinicopathological significance of cytoplasmic and/or nuclear β-catenin expression in patients with HCC. In addition, the association between cytoplasmic and/or nuclear β-catenin expression and etiology (HBV and HCV) was also analyzed.

## Methods

### Study Selection

The Pubmed, Elsevier, Embase, Cochrane Library and Web of Science databases were searched systematically for identified articles published until June 7th, 2014. The terms used in search was as follows:“β-catenin, Beta-catenin, or CTNNB1”, “WNT/β-catenin signal pathway”, "prognostic, prognosis, or survival", “HCC”, “hepatocellular carcinoma”, “hepatic tumor”, “hepatic cancer”, “liver cancer”, “liver tumor” and “liver neoplasms” with all possible combinations. The searched articles were filtered out and the reference lists of eligible ones were taken under scrutiny for additional available studies. The systematic literature search was carried out independently by two reviewers (JC and JL).

### Criteria for Inclusion and Exclusion

To make this meta-analysis meet the high standards, studies had to fulfill the following criteria: (1) patients with distinctive hepatocellular carcinoma diagnosis by pathology but without restriction on age or ethnicity; (2) β-catenin expression was measured by immunohistological chemistry (IHC) or other methods in primary HCC tissue; (3) clinical trials or reports were published in English; (4) sufficient information or valid data about β-catenin expression and OS or RFS and the other clinicopathological features were provided directly or could be calculated indirectly; (5) the study with the highest quality assessment was enrolled when several studies reported by one individual author were conducted on the same patients population; (6) the patients were followed-up for at least 3 years.

Abstracts, editorials, letters and expert opinions, conference records, reviews without original data, case reports and studies lack of control groups were excluded. Studies and data were also excluded if: (1) articles about animals or cell lines; (2) the outcomes or parameters of patients were not clearly reported; (3) no related data required for necessary analysis; (4) overlapping articles.

In this process, the title and abstract of studies were first screened to see whether they met the including criteria. Second, the full text was subjected to further examination following the initial screening. Finally, the eligibility of studies was verified by 2 reviewers (JC and JL) after resolving the disagreements by the third reviewer (RJ).

### Data Extraction and Literature Quality Assessment

Independently, valid data were retrieved from the set of eligible studies by two reviewers (JC and JL) and relevant characteristics were listed as follows: (1) first author and publication year; (2) number of cases; (3) characteristics of subject population, such as age, gender, origin and clinicopthological features; (4) disease stage; (5) methods of evaluating ORs and 95%CI between β-catenin levels and OS or RFS or other clinicopathological parameters; (6) accumulated percentage of β-catenin expression; (7) the number of cases with accumulated β-catenin expression in specific location (Table 1). Any divergence was ironed out by discussion with the third reviewer (RJ) for final expectation of consensus.

**Table 1:** Characteristics of studies included in the meta-analysis.

First author & year		Country or region	No. of patients	Mean age	Gender(M/F)	(C+N)/T	Level of evidence	Stage	Clinicopathological features	Method	antibody source	Dilution	Blind evaluation	Definition standard*	ProvidedOS data	
Jin 2014		Korea	302	54.88(25–77)	254/48	(233+10)/243	5	I–IV	D, T	IHC	NR	NR	Yes	CS	Yes	
Lee 2014		USA	89	51 (20–75)	78/11	(21+8)/29	4	I–IV	D	IHC	(BD Biosciences, San Diego, CA)	1∶50	Yes	CS	NR	
Witjes 2013		Netherlands	47	65 (21–82)	23/24	(0+16)/16	4	NR	D,T	IHC	(DAKO, Japan)	NR	Yes	≥Focal/diffuse	NR	
Geng 2012		China	85	NR	77/8	(52+6)/58	4	I–IV	D,T	IHC	(BD Biosciences, USA)	1∶400	Yes	CS	Yes	
Zhao 2012		China	97	52.86	82/15	(?+?)/66	5	I–IV	D,M	IHC	(Santa Cruz Biotech, USA)	1∶100	Yes	CS	Yes	
Feng 2011		China	63	45.8±10.6 (24–74)	51/12	(35+0)/35	4	NR	D,M	IHC	(BD Biosciences, San Diego, CA)	NR	Yes	>10%cells	NR	
Cheng 2011		Hong Kong	25	47.92(14–72)	23/2	(8+0)/8	3	NR	D	IHC	(DAKO, Japan)	1∶100	Yes	CS	NR	
Liu 2010		China	200	NR	169/31	(?+?)/87	4	I–IIIa	D,T,M	IHC	(BD Biosciences, San Diego, CA)	NR	Yes	Stronger than non-cancerous	Yes	
Zulehner 2010		Austria	133	54.7±9	113/20	(0+78)/78	4	NR	D,M, T	IHC	(Transduction Laboratories, Lexington, UK)	1∶100	Yes	> low staining	NR	
Du 2009		China	43	49(29–72)	36/7	(17+0)/17	3	NR	D,T,M	IHC	(Abgent Biotechnology, CA)	1∶50	Yes	> weak	NR	
Yu 2009		China	314	NR	266/48	(?+?)/126	5	I–III	D,T,M	IHC	(Transduction Laboratories, Lexington, KY)	1∶200	Yes	>10% cells	Yes	
Yang 2009		Taiwan	123	NR	104/19	(?+?)/53	5	I–IV	D,T,M	IHC	(Abcam plc.)	1∶1000	Yes	>10% cells	Yes	
Korita 2008		Japan	125	63(16–79)	88/37	(0+16)/16	3	NR	D,T	IHC	(Novocastra Laboratories Ltd, Newcastleupon-Tyne, United Kingdom)	1∶200	Yes	CS	NR	
Zhai 2008		China	97	54(34–72)	67/30	(36+6)/42	3	I–IV	D,T,M	IHC	(Santa Clauze Corporation, USA)	1∶200	Yes	CS	Yes	
Park 2005		Korea	92	51.6(26–89)	75/17	(?+?)/30	3	I–IV	D	IHC	(Transduction Laboratories, Lexington, Lexington, KY)	NR	Yes	CS	NR	
Tien 2005		Japan	32	64(36–86)	20/8	(7+8)/15	3	NR	D	IHC	(BD Biosciences, San Jose, CA)	1∶200	Yes	Stronger than non-cancerous	NR	
Schmitt Graff 2003		Germany	196	65.3(10.7–86.0)	157/39	(84+73)/157	3	I–IV	NR	IHC	(Transduction Laboratories, Lexington, KY, USA)	1:6000	Yes	≥Focal	Yes	
Inagawa 2002		Japan	51	63.5(45–79)	33/18	(0+18)/18	4	NR	D	IHC	(Transduction Laboratories, Lexington, KY)	1∶200	Yes	Stronger than non-cancerous	NR	
Suzuki 2002		Japan	50	62.4±9.9	38/12	(42+11)/53	3	NR	D,M,T	IHC	(Transduction Laboratories, Lexington, KY)	1∶200	Yes	Stronger than non-cancerous	NR	
Endo 2000		Japan	107	60(17–80)	87/20	(?+?)/84	4	NR	D	IHC	(Transduction Laboratories, Lexington, KY)	1∶100	Yes	CS	Yes	
Huang 1999		Japan/Switzerland	22	62.7±6.3(49–75)	17/5	(0+11)/11	3	NR	D	IHC	(Transduction Laboratories, Lexington, KY)	1∶1000– 2000	Yes	≥ Focal	NR	
Ihara 1996		Japan	41	60.1(42–77)	38/3	(?+?)/58	3	NR	D	IHC	(Transduction Laboratories, Lexington, KY)	10 mg/ml	Yes	CS	NR	

CS: complex score combining intensity and percentage; IHC: immunohistochemistry; D: histologic differentiation degree; T: depth of tumor invasion; M: metastasis; OS: overall survival; NR: not reported; *: The definition standard of β-catenin overexpression in cytoplasm or nucleus; (C+N)/T: the number of tissue samples with β-catenin overexpression in cytoplasm(C) (+) nucleus (N); C: cytoplasm; N: nucleus; T: total, T = C+N; ?: no information was provided.

The quality of each included study was assessed by two reviewers (JC and JL) using Newcastle-Ottawa scale (NOS). NOS evaluated various aspects of methodology, which were grouped into the three dimensions of selection, comparability, and outcome. Final scores ranged from 0 (lowest) to 9 (highest); the higher value, the better eligibility of methodology. The study would not be enrolled in the meta-analysis if its value of quality assessment was too low.

### Statistical Analysis

This meta-analysis was performed using the Review Manager (RevMan) software (version 5.2; Cochrane collaboration) and STATA (version 12.0, Stata Corp. College Station, Texas) [18]. Odds ratios (OR) combined with 95%confidence intervals (CI) was analyzed to evaluate the association of cytoplasmic and/or nuclear β-catenin accumulation with the prognosis and clinicopathological factors of HCCs. Pooled values of ORs and 95%CIs serve as the recommended summary statistics for meta-analysis of β-catenin expression on prognostic value and clinicopathological features, such as OS, RFS, differentiation grade, TNM stages, metastasis, vascular invasion and liver cirrhosis. In some studies, these statistical variables were depicted in original studies, and we pooled them directly; otherwise, indirectly, we obtained these variables from the available data or by reading Kaplan-Meier survival curve according to the method described by Parmar MK, which has been widely applied in meta-analysis about prognosis [19]. Heterogeneity among the outcomes of enrolled studies in this meta-analysis was evaluated by using Chi-square based Q statistical test [20]. And *I* ^2^ statistic, ranging from 0% to 100%, was used to estimate the proportion of total variation caused by inter-study heterogeneity (*I* ^2^ = 0-50%, no or moderate heterogeneity; *I* ^2^>50%, significant heterogeneity). By heterogeneity test, according to the result of Q statistical test, a fixed-effects model was selected if *P*>0.05 and a random-effects model was selected if *P*<0.05. *P*<0.05 was considered statistically significant. The funnel plots were made by utilizing Egger's test and Begg's test to examine the risk of potential publication bias. Then, trim and fill analyses were used to evaluate the stability of our meta-analysis results if the plots were asymmetrical.

## Results

### Selection of Trials

**Figure 1:**
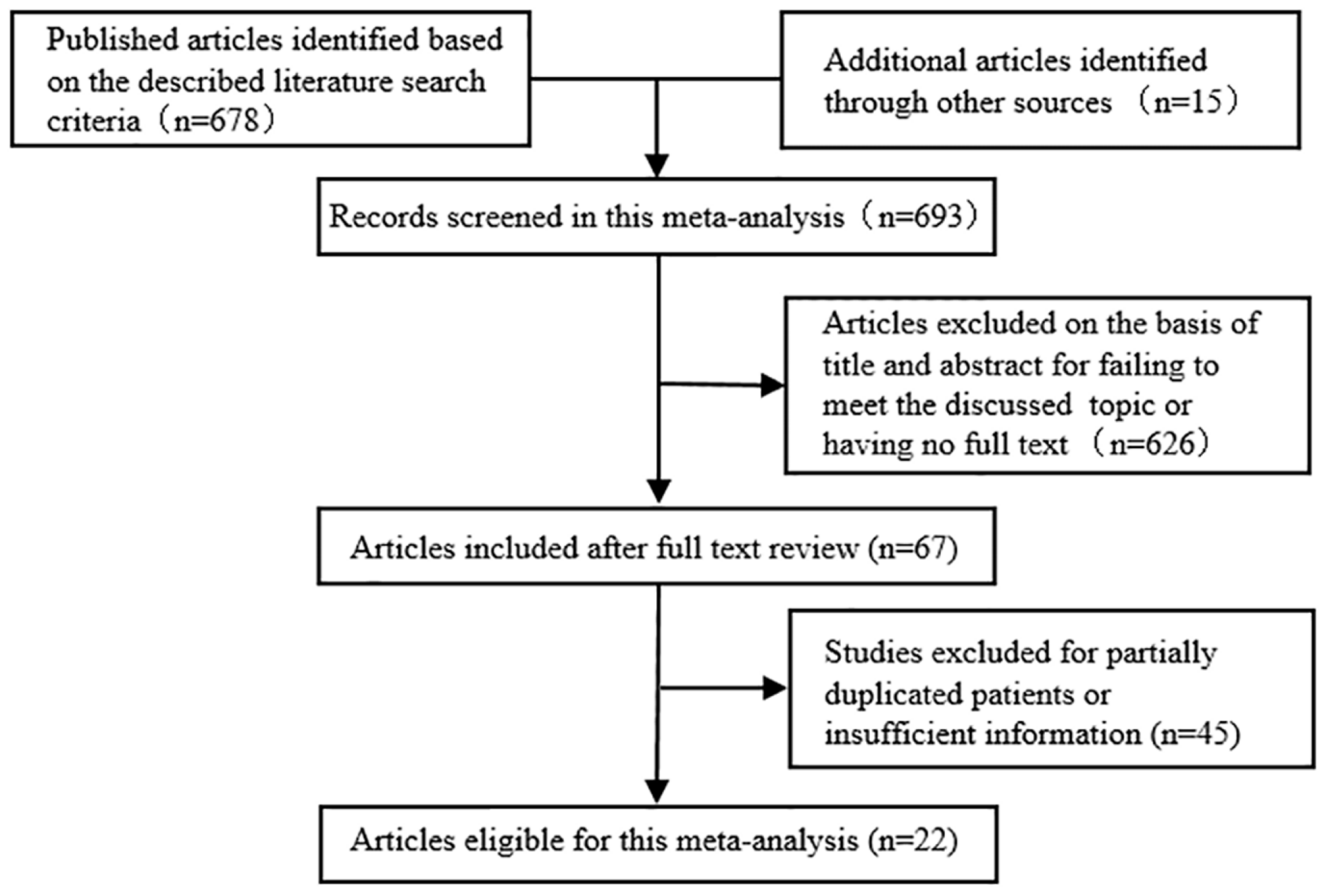
Flow chart of literature search strategies.

As shown in Figure 1, total 693 potentially eligible studies were screened out in the preliminary search. And then 626 studies were excluded because their titles or abstract failed to meet the discussed topic or these studies had no full text. 67 full papers were captured, among which 45 studies were ultimately excluded due to the lack of adequate data on β-catenin expression level and specific parameters. Thus, 22 studies, with more detailed and sufficient evaluation, met our entry criteria and were retrieved for further analysis. The flow diagram of literature selection procedure was depicted in Figure1. The major clinical characteristics of these enrolled studies are outlined in Table 1. The total number of cases was 2334. The cases size ranged from 22 to 302 patients. Among these cases, 1853 tissue samples showed cytoplasmic and/or nuclear accumulation of β-catenin. The data about status of accumulated β-catenin expression, prognostic value, pathological features and etiology had been extracted from these cases. Cytoplasmic and/or nuclear expression rate of β-catenin had a wide range due to the limited number of cases and the circumscribed region of subjects in each study. Subgroup analysis by grouping basing on β-catenin expression rate (≥50% vs <50%) was performed as shown in Table 2. Most of the studies enrolled in this meta-analysis were performed in Asian population. Subgroup analysis by origin (Asia vs others) was conducted in this meta-analysis as shown in Table 3. The ORs and 95%CI between β-catenin expression and overall survival were provided directly or calculated indirectly. Just as shown in Table 1, all studies enrolled in this meta-analysis were performed properly and the expression of β-catenin in specific location (cytomembrane or cytoplasm or nucleus) was determined by using immunohistochemistry without subjective interference.

**Table 2:** Subgroup analysis for prognostic and clinicopathological significance of cytoplasmic and/or nuclear β-catenin expression by its rate (≥50% vs <50%).

Factors	Positive expression rate	Number of studies	OR	95%CI	Z	P	I^2^
1-year OS	≥50%	5	0.62	0.38–1.00	1.95	0.05	4
	<50%	4	0.58	0.31–1.08	1.72	0.09	6
3-year OS	≥50%	5	0.43	0.21–0.91	2.22	0.03	64
	<50%	4	0.75	0.30–1.84	0.63	0.53	79
5-year OS	≥50%	5	0.36	0.19–0.69	3.11	0.002	54
	<50%	4	0.80	0.22–2.94	0.34	0.73	88
1-year RFS	≥50%	1	0.38	0.14–1.01	1.93	0.05	-
	<50%	3	0.58	0.25–1.35	1.26	0.21	62
3-year RFS	≥50%	1	0.41	0.18–0.93	2.14	0.03	-
	<50%	3	0.35	0.24–0.49	5.86	<0.00001	0
5-year RFS	≥50%	1	0.37	0.16–0.83	2.42	0.02	-
	<50%	3	0.34	0.24–0.49	5.83	<0.00001	
Metastasis	≥50%	2	0.62	0.30–1.28	1.29	0.2	0
	<50%	6	0.64	0.42–0.97	2.09	0.04	0
Vascular invasion	≥50%	2	0.30	0.14–0.62	3.26	0.001	0
	<50%	5	0.50	0.33–0.78	3.08	0.002	0

**Table 3:** Subgroup analysis for prognostic and clinicopathological significance of cytoplasmic and/or nuclear β-catenin expression by origin (Asia vs others) and level of evidence (≥4 vs <4).

Factors	subgroup		Number of studies	OR	95%CI	Z	P	I^2^
1-year OS	Origin							
		Asia	8	0.49	0.30-0.77	3.05	0.002	0
		others	1	1.01	0.50–2.06	0.04	0.97	-
	Level of evidence							
		≥4	7	0.49	0.30–0.77	3.05	0.002	0
		<4	2	1.01	0.50–2.06	0.04	0.97	-
3-year OS	Origin							
		Asia	8	0.5	0.28–0.89	2.35	0.02	70
		others	1	1.24	0.52–2.92	0.49	0.63	-
	Level of evidence							
		≥4	7	0.43	0.32–0.57	5.58	<0.00001	45
		<4	2	1.75	0.92–3.30	1.72	0.09	25
5-year OS	Origin							
		Asia	8	0.41	0.21–0.81	2.57	0.01	77
		others	1	0.99	0.40–2.48	0.02	0.99	-
	Level of evidence							
		≥4	7	0.33	0.19–0.56	4.07	<0.0001	65
		<4	2	6.22	0.07–534.37	0.8	0.42	89
1-year RFS	Origin							
		Asia	4	0.47	0.32–0.70	3.68	0.0002	44
		others	0	-	-	-	-	-
	Level of evidence							
		≥4	4	0.47	0.32–0.70	3.68	0.0002	44
		<4	0	-	-	-	-	-
3-year RFS	Origin							
		Asia	4	0.36	0.26–0.49	6.23	<0.00001	0
		others	0	-	-	-	-	-
	Level of evidence							
		≥4	4	0.36	0.26–0.49	6.23	<0.00001	0
		<4	0	-	-	-	-	-
5-year RFS	Origin							
		Asia	4	0.35	0.25–0.48	6.31	<0.00001	0
		others	0	-	-	-	-	-
	Level of evidence							
		≥4	4	0.35	0.25–0.48	6.31	<0.00001	0
		<4	0	-	-	-	-	-
metastasis	Origin							
		Asia	7	0.65	0.45–0.96	2.19	0.03	0
		others	1	0.44	0.11–1.72	1.18	0.24	-
	Level of evidence							
		≥4	6	0.58	0.39–0.87	2.63	0.009	0
		<4	2	0.92	0.40–2.12	0.2	0.84	0
Vascular invasion	Origin							
		Asia	5	0.46	0.30–0.72	3.38	0.0007	4
		others	2	0.38	0.19–0.75	2.8	0.005	0
	Level of evidence							
		≥4	5	0.35	0.23–0.54	4.68	<0.00001	0
		<4	2	0.86	0.41–1.82	0.39	0.7	0

### Quality Assessment

Methodological quality of the 22 studies was assessed according to NOS. The results of quality assessment were shown in the ‘level of evidence’ column of Table 1. Of the 22 studies, 4 ones [21]–[24] scored 5 points, 8 [15], [25]–[31] scored 4, 10 [32]–[41] scored 3. Subgroup analysis by level of evidence (≥4 vs <4) was conducted to investigate if studies with higher or lower level of evidence could make differences in results for prognostic value and clinicopathological significance of cytoplasmic and/or nuclear β-catenin expression. Studies with score of 5 or more were regarded as high quality. It indicated that 4 studies obtaining score of 5 in methodological assessment were of high quality.

### Impact of Cytoplasmic and/or Nuclear B-Catenin Expression on OS and RFS

**Figure 2:**
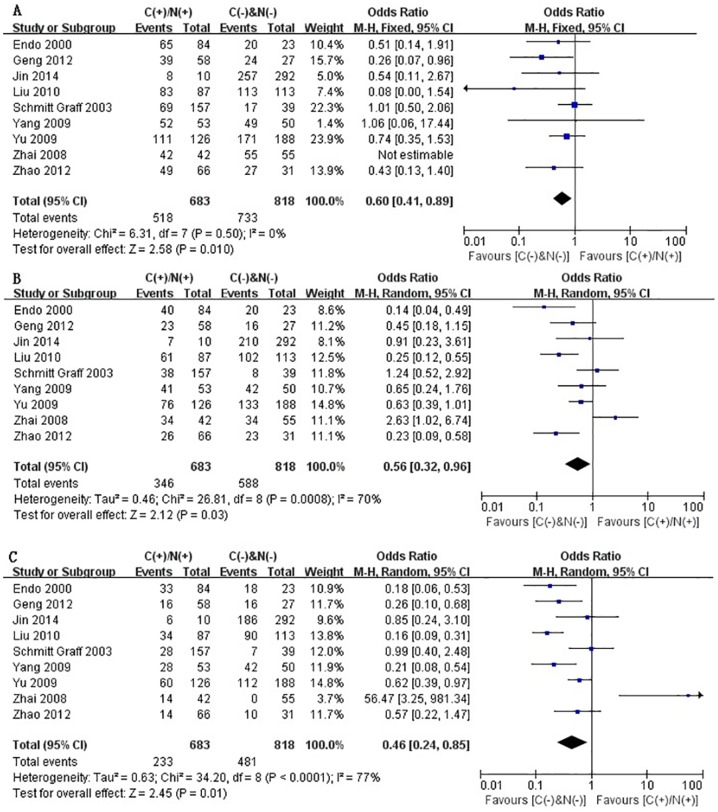
Forest plot of odds ratio for the association of β-catenin expression in cytoplasm and/or nucleus with 1-year (A), 3-year (B) and 5-year (C) overall survival.

**Figure 3:**
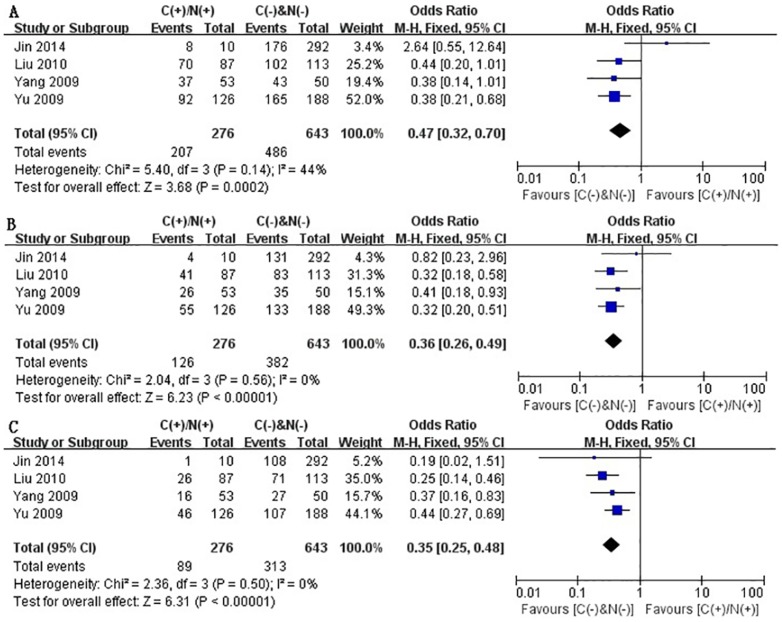
Forest plot of odds ratio for the association of β-catenin expression in cytoplasm and/or nucleus with1-year (A), 3-year (B) and 5-year (C) recurrence-free survival.

Some of the enrolled studies provided the ORs and 95%CI directly or indirectly when they investigated the correlation between cytoplasmic and/or nuclear β-catenin expression and overall survival (OS) or recurrence-free survival (RFS) (Table 4). By pooling these relevant data, we systematically assessed the association of cytoplasmic and/or nuclear expression of β-catenin with OS and RFS of patients with HCC by phasing three periods, one-year, three-year and five-year, respectively. On the basis of 9 retrospective studies [21]–[24], [28], [30], [31], [39], [41], it was found that aberrant accumulation of β-catenin in cytoplasm or nucleus significantly correlated with poor 1-, 3- and 5-year OS, just as shown in Fig.2 (A–C). The pooled ORs were 0.60 (n = 9 studies, 95% CI: 0.41–0.89, Z = 2.58, P = 0.010), 0.56 (n = 9 studies, 95% CI: 0.32–0.96, Z = 2.12, P = 0.03) and 0.46 (n = 9 studies, 95% CI: 0.24–0.85, Z = 2.45 P = 0.010) and statistical heterogeneity was 0%, 70% and 77%, respectively for 1-, 3- and 5-year OS. In Fig.3 (A–C), the RFS correlated with cytoplasmic and/or nuclear overexpression of β-catenin was evaluated basing on 4 studies [21], [23], [24], [28], the combined ORs were 0.47 (n = 4 studies, 95% CI: 0.32–0.70, Z = 3.68, P = 0.0002), 0.36 (n = 4 studies, 95% CI: 0.26–0.49, Z = 6.23, P<0.00001) and 0.35 (n = 4 studies, 95% CI: 0.25–0.48, Z = 6.31, P<0.00001), respectively. And no significant heterogeneity was observed (*I* ^2^ = 0% for 1-, 3- and 5-year RFS). Taken together, the above results suggested that β-catenin accumulation in cytoplasm and/or nucleus was significantly correlated with a worse prognosis of HCC. That is to say, patients with HCC showing no β-catenin overexpression in cytoplasm and nuclear were found to have a better prognosis than those patients with HCC showing cytoplasmic and/or nuclear β-catenin overexpression.

**Table 4:** Results of a meta-analysis comparing HCC with C/N β-catenin expression to M β-catenin expression.

Outcome of interest	No. of studies	Number of tissue samples	OR/WMD	95% CI	P value	I^2^ (%)
Overall Survival						
1 year	9	C(+)/N(+) = 683, C(-)&N(-) = 818	0.60	0.41–0.89	0.01	0
3 year	9	C(+)/N(+) = 683, C(-)&N(-) = 818	0.56	0.32–0.96	0.03	70
5year	9	C(+)/N(+) = 683, C(-)&N(-) = 818	0.46	0.24–0.85	0.01	77
Recurrence-free Survival						
1 year	4	C(+)/N(+) = 276, C(-)&N(-) = 643	0.47	0.32–0.70	= 0.0002	44
3 year	4	C(+)/N(+) = 276, C(-)&N(-) = 643	0.36	0.26–0.49	<0.00001	0
5 year	4	C(+)/N(+) = 276, C(-)&N(-) = 643	0.35	0.25–0.48	<0.00001	0
Differentiation grade	16	C(+)/N(+) = 627, C(-)&N(-) = 734	1.24	0.71–2.19	0.45	60
Metastasis	8	C(+)/N(+) = 347, C(-)&N(-) = 646	0.63	0.44–0.91	0.01	0
-Subgroup 1	4	N(+) = 117, N(-) = 419	0.66	0.34–1.25	0.20	0
-Subgroup 2	3	C(+) = 310, C(-) = 105	0.93	0.56–1.55	0.79	79
Vascular invasion	7	C(+)/N(+) = 337, C(-)&N(-) = 485	0.44	0.30–0.63	<0.0001	0
- Subgroup 1	4	N(+) = 115, N(-) = 420	0.49	0.28–0.84	0.009	0
- Subgroup 2	2	C(+) = 275, C(-) = 77	0.70	0.42–1.17	0.18	0
TNM stage	7	C(+)/N(+) = 300, C(-)&N(-) = 712	1.18	0.81–1.71	0.39	41
Tumor encapsulation	3	C(+)/N(+) = 179, C(-)&N(-) = 305	1.25	0.64–2.43	0.52	51
Liver cirrhosis	3	C(+)/N(+) = 215, C(-)&N(-) = 307	1.59	0.63–3.97	0.33	51
Tumor size	6	C(+)/N(+) = 260, C(-)&N(-) = 446	1.00	0.49–2.01	0.99	66
HBV	6	C(+)/N(+) = 269, C(-)&N(-) = 349	1.17	0.78–1.76	0.45	0
HCV	4	C(+)/N(+) = 147, C(-)&N(-) = 208	0.52	0.25–1.09	0.08	4
AFP	4	C(+)/N(+) = 214, C(-)&N(-) = 335	0.85	0.59–1.22	0.39	44

C(+)/N(+): β-catenin expression in cytoplasm(C) and/or nucleus (N); C(-)&N(-): none β-catenin expression in cytoplasm(C) and nucleus (N); OR: odds ratio; WMD: weighted mean difference; CI: confidence interval.

### Correlation of Cytoplasmic and/or Nuclear B-Catenin Expression with Clinicopathological Parameters

**Figure 4:**
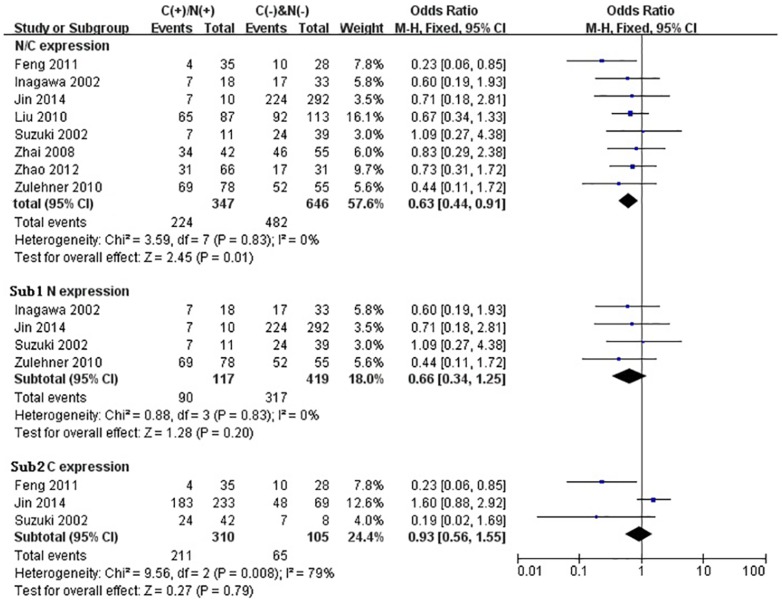
Forest plot of odds ratio for the association of β-catenin expression in cytoplasm and/or nucleus with metastasis by subgroup analysis. Sub1: in cytoplasm alone. Sub2: β-catenin expression in nucleus alone.

**Figure 5:**
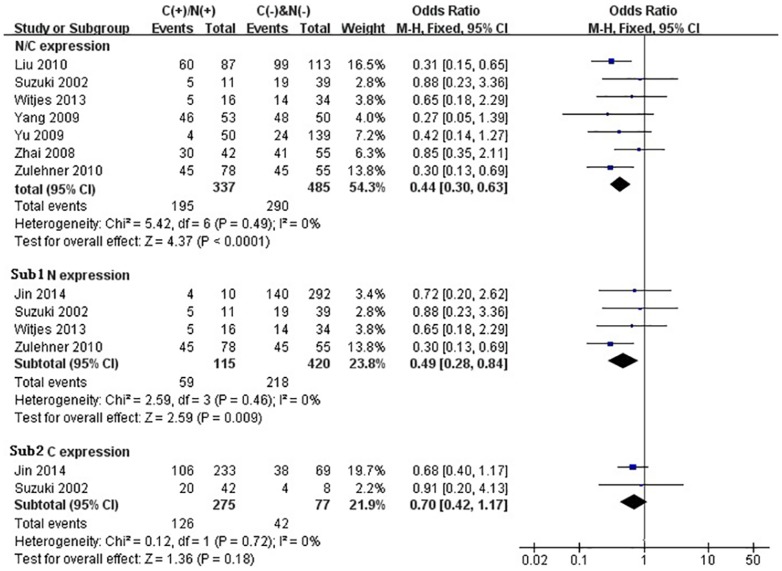
Forest plot of odds ratio for the association of β-catenin expression in cytoplasm and/or nucleus with vascular invasion by subgroup analysis. Sub1: in cytoplasm alone. Sub2: β-catenin expression in nucleus alone.

**Figure 6:**
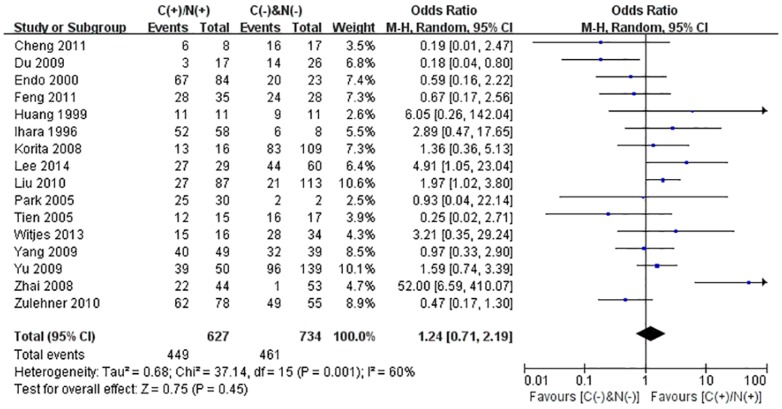
Forest plot of odds ratio for the association of β-catenin expression in cytoplasm and/or nucleus with differentiation grade.

**Figure 7:**
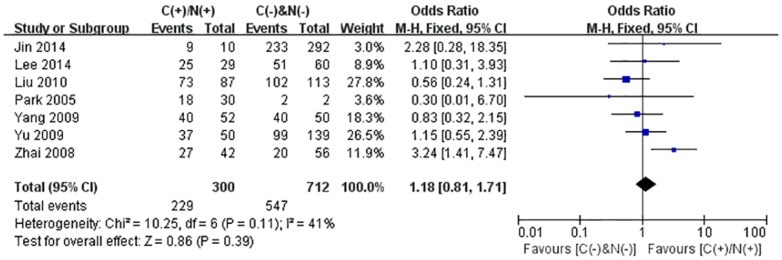
Forest plot of odds ratio for the association of β-catenin expression in cytoplasm and/or nucleus with TNM stage.

**Figure 8:**
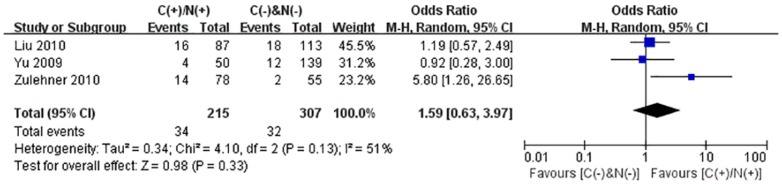
Forest plot of odds ratio for the association of β-catenin expression in cytoplasm and/or nucleus with liver cirrhosis.

**Figure 9:**
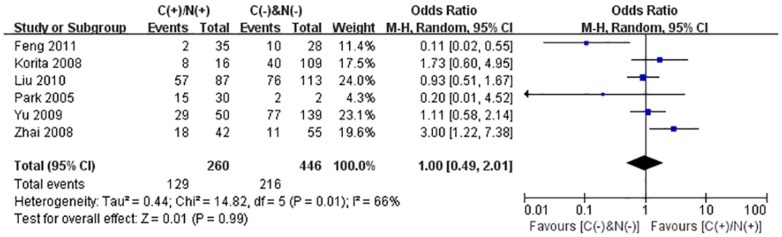
Forest plot of odds ratio for the association of β-catenin expression in cytoplasm and/or nucleus with tumor size.

**Figure 10:**
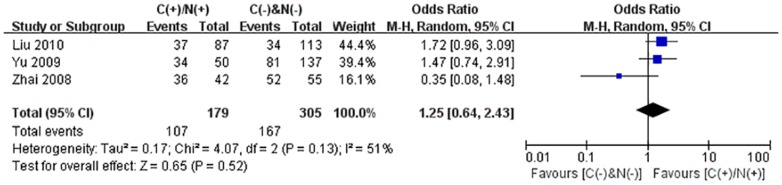
Forest plot of odds ratio for the association of β-catenin expression in cytoplasm and/or nucleus with Tumor encapsulation.

**Figure 11:**
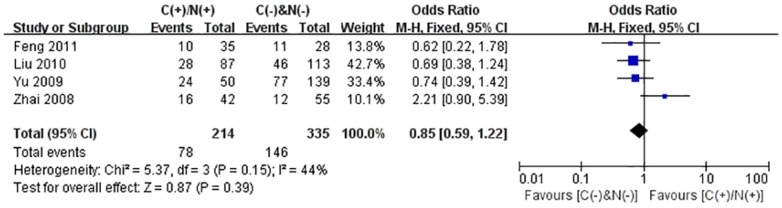
Forest plot of odds ratio for the association of β-catenin expression in cytoplasm and/or nucleus with AFP level.

We performed the meta-analysis to evaluate the correlation between β-catenin expression in cytoplasm and/or nucleus with clinicopathological parameters, namely, metastasis, vascular invasion, differentiation grade, TNM stages, liver cirrhosis, tumor size, tumor encapsulation and alpha fetal protein (AFP). Eight studies [21], [22], [26]–[29], [39], [40] investigated the association of cytoplasmic and/or nuclear expression of β-catenin with metastasis of HCC (Fig.4a). The combined OR was 0.63(95%CI: 0.44–0.91, Z = 2.45, P = 0.01) and the statistic heterogeneity is not significant (*I^2^* = 0%). This result suggested that there was significant correlation between cytoplasmic and/or nuclear accumulation of β-catenin and metastasis of HCC. As depicted in Fig.4b–c, subgroup analysis was also conducted with expectation to further investigate the effect of specific location (cytoplasm or nucleus) of β-catenin accumulation on metastasis. The results showed that nuclear or cytoplasmic overexpression alone was not significantly correlated with metastasis. The pooled ORs were 0.66(n = 4, 95%CI: 0.34–1.25, Z = 1.28, P = 0.20) for the former and 0.93(n = 3, 95%CI: 0.56–1.55, Z = 0.27, P = 0.79) for the latter. Seven studies [23]–[25], [27], [28], [39], [40] assessed the relationship of aberrant accumulation of β-catenin in cytoplasm and/or nucleus with vascular invasion with no heterogeneity (Fig.5a). The pooled OR was 0.44 (95%CI: 0.30–0.63, Z = 4.37, P<0.0001), indicating that β-catenin accumulation in cytoplasm and/or nucleus was closely correlated with vascular invasion. And then subgroup analysis were also performed just as shown in Fig.5b–c. The results denoted that nuclear overexpression alone had a more close relationship with vascular invasion than cytoplasmic overexpression alone. It indicated that nuclear overexpression alone or combined with cytoplasmic overexpression significantly correlated with vascular invasion. Additionally, we also assessed the relationship of cytoplasmic and/or nuclear β-catenin overexpression with differentiation grade (III/IV versus I/II) and TNM stages (T3/T4 versus T1/T2) on the basis of sixteen studies [15], [23]–[28], [30], [32]–[39] and seven studies [15], [23], [24], [28], [36], [39], [42], respectively (Table 4). It was found that β-catenin accumulation in cytoplasm and/or nucleus had no worse effect on the two clinicopathological features. As shown in Fig.6 and Fig.7, the combined ORs were 1.24 (95% CI: 0.71–2.19, Z = 0.75, P = 0.45) and 1.18 (95% CI: 0.81–1.71, Z = 0.86, P = 0.39) and statistic heterogeneity was 60% and 41%. For other clinicopathological parameters, such as liver cirrhosis (Fig.8), tumor size (Fig.9), tumor encapsulation (Fig.10) and AFP level (Fig.11), of HCC showing accumulated expression of β-catenin in cytoplasm and/or nucleus, the pooled ORs were 1.59 (n = 3 studies, 95%CI: 0.63–3.97, Z = 0.98, P = 0.33), 1.00 (n = 6 studies, 95%CI: 0.49–2.01, Z = 0.01, P = 0.99), 1.25 (n = 3 studies, 95%CI: 0.64–2.43, Z = 0.65, P = 0.52) and 0.85 (n = 4 studies, 95%CI: 0.59–1.22, Z = 0.87, P = 0.39) indicating that cytoplasmic and/or nuclear accumulation of β-catenin had no significant correlation with these parameters.

### Correlation of Cytoplasmic and/or Nuclear B-Catenin Expression with Etiology

**Figure 12:**
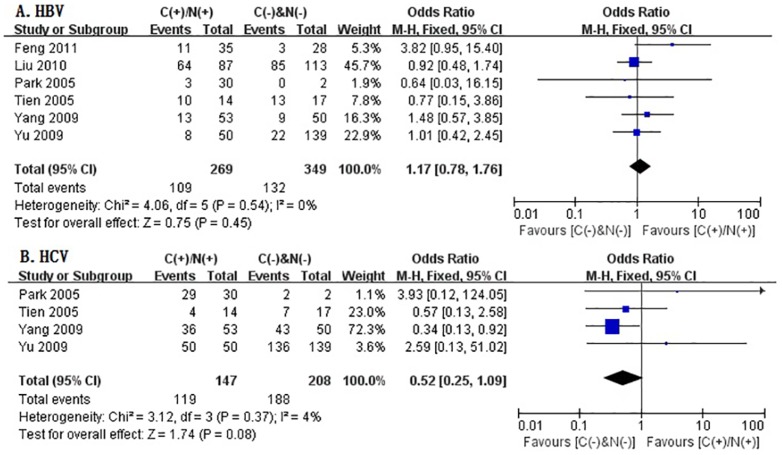
Forest plot of odds ratio for the association of β-catenin expression in cytoplasm and/or nucleus with etiology. **A: HBV; B: HCV.**

In this meta-analysis, the correlation between cytoplasmic and/or nuclear β-catenin expression and etiology (HBV and HCV) was evaluated basing on the retrospective studies which provided the relevant data. Just as depicted in Fig.12A–B, six studies assessed the association of β-catenin overexpression in cytoplasm and/or nucleus with HBV, and four studies investigated the relationship between β-catenin overexpression in cytoplasm and/or nucleus and HCV. The combined ORs were 1.17 (95%CI: 0.78–1.76, Z = 0.75, P = 0.45) for the former and 0.52(95%CI: 0.25–1.09, Z = 1.74, P = 0.08) for the latter. The statistic heterogeneity was not significant (*I* ^2^ = 0%, 4%, respectively). The results suggested that no significant correlation was observed between cytoplasmic and/or nuclear β-catenin expression and etiology (HBV and HCV).

### Subgroup Analysis by Grouping Basing on B-Catenin Expression Rate, Origin and Level of Evidence

To critically investigate prognostic and clinicopathological significance of cytoplasmic and/or nuclear β-catenin expression, subgroup analysis by positive β-catenin expression rate was performed and the results showed that in the group of studies with higher aberrant β-catenin expression rate, a more significant correlation was observed between cytoplasmic and/or nuclear β-catenin expression and poor prognosis (Table 2). The detailed information was presented in Figure S1, S2, S3, S4, S5, S6, S7, S8 in File S1. However, lower aberrant expression rate of β-catenin (<50%) tended to barely affect the relationship between cytoplasmic and/or nuclear β-catenin expression and prognostic value. It indicated that different positive β-catenin expression rate of all tissue samples in each study would make differences in results for the correlation between cytoplasmic and/or nuclear β-catenin expression and prognostic value. Different sample size and wide-scope expression rate involved in each study may induce potential risks of weakening the results of large sample with better quality and strengthening the effect of the small sample with worse quality. In Table 3, the results of subgroup analysis by origin showed that Asian population exhibited a more close association between cytoplasmic and/or nuclear β-catenin expression and poor prognosis and unfavorable clinicopathological factors. In other origins, mainly western population, no significant correlation was observed between cytoplasmic and/or nuclear β-catenin expression and prognostic and clinicopathological value. But the number of studies conducted in western populations was very limited and the limited number may result in no significant changes. Another subgroup analysis was performed by grouping basing on level of evidence (≥4 vs <4), as depicted in Table 3. The results suggested that in the group of studies with higher level of evidence, cytoplasmic and/or nuclear β-catenin expression showed a more robust correlation with poor prognosis and clinicopathological significance. Studies with lower level of evidence usually provided comparatively less adequate data and thus had no advantages during evaluating prognostic value and clinicopathological significance of cytoplasmic and/or nuclear β-catenin expression.

### Multivariate Analyses of Factors Associated with Survival and Recurrence

In Table 5, multivariate analyses were performed to determine if cytoplasmic and/or nuclear β-catenin expression has an independent prognostic value compared to other clinical and pathological features, such as metastasis, vascular invasion, differentiation grade, TNM stage, liver cirrhosis, tumor size, tumor encapsulation and AFP level. The results showed that β-catenin overexpression in cytoplasm and/or nucleus, as well as metastasis, TNM stage and tumor size, was an independent prognostic factor. It implied that β-catenin could serve as a novel independent target of developing clinical therapies for patients with HCC.

**Table 5:** Multivariate analyses of factors associated with survival and recurrence.

Factors	Overall survival			Recurrence free survival		
	HR	95%CI	P value	HR	95%CI	P value
HBeAg (positive vs negative)	0.89	0.54–1.45	0.63	1.36	0.88–2.12	0.17
Liver cirrhosis (no vs yes)	2.02	1.00–4.09	0.17	1.55	0.86–2.80	0.15
AFP level (<20 ng/mL vs ≥21 ng/mL)	1.54	1.10–2.16	0.01	1.29	0.61–2.72	0.51
Tumor differentiation (I–II vs III–IV)	1.34	0.90–1.99	0.15	1.08	0.87–1.34	0.48
Tumor size, (≤5 cm vs >5 cm)	1.08	1.03–1.12	0.001	1.06	1.01–1.10	0.008
Metastasis (no vs yes)	1.36	1.16–1.61	0.0002	1.32	1.15–1.51	<0.0001
Tumor encapsulation (complete vs none)	0.99	0.59–1.69	0.99	0.86	0.56–1.33	0.50
Vascular invasion (no vs yes)	1.53	0.94–2.48	0.09	1.23	0.71–2.12	0.45
TNM stage(I/II vs III/IV)	1.68	1.27–2.22	0.0003	1.67	1.29–2.15	<0.0001
β-catenin (C(+)/N(+) vs C(-)&N(-))	2.73	2.03–3.66	<0.00001	2.56	1.53–4.28	0.0003

### Publication Bias

Begg's test indicated that there was no evidence of significant publication bias after assessing the funnel plot (Figure S9, S10, S11, S12, S13, S14, S15, S16, S17, S18, S19 in File S1) for the studies included in our meta-analysis.

## Discussion

Hepatocellular carcinoma (HCC) is the third most fatal cancer worldwide and a major health threat [21]. Its high morbidity and mortality makes understanding its cellular and molecular basis and broadening the currently limited treatment options in urgent need [16], [21]. The WNT/β-catenin pathway had been well-studied and proven to be involved in progression of several tumors. B-Catenin is a double-functional molecule. In addition to its role in intercellular adhesion, it can also serve as a key downstream effector and a crucial signaling molecule in WNT signaling pathway. In malignant hepatocytes, β-catenin loses its function as a cell-adhesion molecule, accumulates in the cytoplasm, translocates to the nucleus, activates the WNT signaling pathway and switches on transcription of target genes such as c-*myc* or cyclin D1, resulting in proliferation and metastasis of tumor cells. So far, tremendous work has been done dedicated to investigating the relationship between cytoplasmic and/or nuclear β-catenin expression and the prognostic and clinicopathological value of patients with HCC but no conclusive result was achieved. On the other hand, meta-analytical technique, as a useful tool in clinical researches, has been utilized commonly to evaluate the value of prognostic predictors in different clinical trials. Its evaluation was systematically qualitative and quantitative. It can realize the consensus for those subjects still with controversial results by integrating and comparing these results to estimate the outcome of interests. Therefore, we investigated 2334 cases extracted from 22 enrolled studies to conduct a systematic and comprehensive meta-analysis to address the association between cytoplasmic and/or nuclear accumulation of β-catenin and prognosis and clinicopathological factors of patients with HCC. And this effort will also be dedicated to identify β-catenin as a novel independent target of developing clinical therapies for patients with HCC.

Despite new therapies of HCC arising continually, researchers still felt gloomy for its poor prognosis mainly caused by metastasis and recurrence. That's why many researchers have been dedicated to finding out more reliable and exact predicators of prognosis for patients with HCC. The β-catenin protein has been reported to be located at the cell membrane, in the cytoplasm, or in the nucleus [12]. In inactivated cells, the majority of β-catenin is mainly located in the membrane and integrated into adhesion complex responsible for the maintenance of cell junctions and the rest β-catenin, free in cytoplasm, is bound to destruction complex and degraded. Once WNT/β-catenin signaling pathway aberrantly activated, membranous expression of β-catenin is reduced and cytoplasmic degradation of β-catenin is prevented, allowing free β-catenin to accumulate in cytoplasm and translocate to the nucleus, where it interacts with transcription factors of the TCF and LEF family (TCF/LEF) to regulate various target genes [43]. Many researchers have reported that β-catenin overexpression in cytoplasm and/or nucleus is closely correlated with metastasis and poor prognosis [31]. But other researchers put forward different points suggesting that no significant correlation was found between β-catenin accumulation in cytoplasm and/or nucleus and prognosis [24], [39]–[41]. In this meta-analysis, a systematic and comprehensive analysis was performed and the result denoted that cytoplasmic and/or nuclear accumulation of β-catenin had worse impact on OS and RFS. Additionally, it was found that nuclear combined with cytoplasmic overexpression of β-catenin significantly correlated with metastasis and vascular invasion. However, no significant correlation was observed between cytoplasmic and/or nuclear β-catenin accumulation and other clinicopathological characteristics, such as differentiation grade, TNM stages, liver cirrhosis, tumor size, tumor encapsulation and AFP level. Besides, β-catenin accumulation in cytoplasm and/or nucleus showed no significant association with HBV and HCV. Moreover, multivariate analyses suggested that cytoplasmic and/or nuclear β-catenin expression was a significantly independent prognostic factor. Taken together, all above results suggested that cytoplasmic and/or nuclear expression of β-catenin, as an independent factor, closely correlated with dismal prognosis, disease development and deeper invasion of HCC. The accumulation of β-catenin in cytoplasm and/or nucleus could serve as a biomarker to predict prognostic value and clinicopathological significance of HCC and act as a target of newly developed therapies.

Although we obtained substantial results in this meta-analysis, no more significant conclusions could be reached due to the weakness of the enrolled studies. It was clearly shown from taxonomic studies that different clusters of HCCs with WNT activation can be split depending on CTNNB1 mutation or not. However, the enrolled studies in this meta-analysis failed to provide adequate information about relationship between cytoplasmic and/or nuclear β-catenin expression and CTNNB1 mutation. Thus, we couldn't investigate their further relationship by grouping depending on CTNNB1 mutation or not. What's more, during the process of our study selection, we learned that tremendous studies have been done about CTNNB1 mutation and WNT pathway but only a very limited number of clinical studies reported the qualitative relationship between CTNNB1 mutation and its prognostic value. Of the total 22 studies, only 2 ones [36], [37] provided the qualitative clinical data. Therefore, we have to screen studies according to cytoplasmic and/or nuclear β-catenin expression though screening basing on CTNNB1 mutation seems more reliable. Moreover, the studies included in this meta-analysis failed to provide symmetric information about location and accumulated percentage of β-catenin expression and prognosis value. Just as depicted in the fifth column of Table 1, some studies provided the number of tissue samples with β-catenin overexpression located in cytoplasm and nucleus respectively but failed to provide the information of prognostic value corresponding to different locations. Some studies only provided the total number of tissue samples with β-catenin overexpression in cytoplasm and/or nucleus but failed to provide the information about locations in detail. Therefore, it was difficult to evaluate the prognostic value of cytoplasmic or nuclear expression alone by pooling data extracted from each enrolled study in this meta-analysis. We know that nuclear β-catenin expression is clearly linked with activation of the WNT pathway and has significant effect on prognosis but cytoplasmic β-catenin expression alone is not strictly correlated with WNT activation and its prognostic value is inconclusive among researchers. Feng et al [26] and Suzuki et al [40] had reported that cytoplasmic β-catenin expression alone led to metastasis and deeper invasion while Jin et al [21] reported that no changes were observed when β-catenin was ectopically expressed in cytoplasm alone. Therefore, for those included studies combining cytoplasmic with nuclear expression of β-catenin, their conclusion about prognostic values could be bias. Inevitably, the conclusion of this meta-analysis based on these studies could be bias, too. In addition, most of the studies included in this meta-analysis were performed in Asian population, so it was not clear if the results of this meta-analysis will be validated in a western population with HCC. Besides, in this meta-analysis, we mainly analyzed tumors surgically resected rather than palliative HCCs or HCC treated by liver transplantation or radiofrequency ablation, considering the limited retrospective studies, poor representation and thereby inaccuracy of the latter.

In addition, in this meta-analysis, there also remained some limitations worthy of further concern. First, heterogeneity was inevitable among the groups due to differences of patient characteristics such as age, gender and chemotherapies in each study. On the one hand, we used a random-effects model in order to eliminate variations across studies. On the other hand, sensitivity analysis was also used to observe whether omission of any single study would have significant impact on the combined OR estimates. Although the effort could not necessarily rule out the effect of heterogeneity among studies, the adverse influence will be weakened to some degree. Second, bias was unavoidable for clinical evidence because some relevant data were retrieved indirectly from several studies or by reading the survival curve. The inaccurate reading might produce errors and bring about bias. Third, studies performed with positive results or significant outcomes will be apt to be published, suggesting a potential publication bias. Fourth, reports in other languages than English were excluded, so potential language bias may be present in our meta-analysis. Fifth, a significant heterogeneity might also be brought about in this meta-analysis by the difference of the antibodies used for test of ectopic cytoplasmic/nuclear β-catenin expression. Besides, the difference of sample size in each study may induce potential risks of weakening the results of large sample with better quality and strengthening the effect of the small sample with worse quality. All these bias or risks should not be neglected and further investigation should be given to determine whether these factors influence the results of the meta-analysis.

The prognostic and clinicopathological significance of β-catenin expression in cytoplasm or nucleus for patients with HCC was investigated in this meta-analysis. The result suggested that patients with HCC showing cytoplasmic and/or nuclear expression of β-catenin appeared to have a poorer OS and RFS, in comparison with those patients with HCC showing normal membranous expression of β-catenin. Additionally, it showed that nuclear combined with cytoplasmic accumulation of β-catenin significantly correlated with metastasis and vascular invasion, while no significant relationship was observed between β-catenin overexpression in cytoplasm and/or nucleus and advanced differentiation grade, TNM stage, liver cirrhosis, tumor size, tumor encapsulation and AFP level. Besides, no significant correlation was observed between cytoplasmic and/or nuclear β-catenin expression and etiology (HBV and HCV). Moreover, cytoplasmic and/or nuclear β-catenin expression was an independent prognostic factor. In summary, β-catenin accumulation in cytoplasm and/or nucleus, as an independent prognostic factor, closely associated with poor prognosis and deep invasion for patients with HCC. Thus, cytoplasmic and/or nuclear expression of β-catenin could serve as a potential predictor for progression and prognosis of patients with HCC and act as a novel target of the developed therapies.

## Supporting Information

Checklist S1 PRISMA checklist
(DOC)

File S1 **Supporting figures.** Figure S1, Forest plot of odds ratio for the association of β-catenin expression in cytoplasm and/or nucleus with 1-year overall survival by subgroup analysis. Sub1: ≥50% expression rate; Sub 2: <50% expression rate. Figure S2, Forest plot of odds ratio for the association of β-catenin expression in cytoplasm and/or nucleus with 3-year overall survival by subgroup analysis. Sub1: ≥50% expression rate; Sub 2: <50% expression rate. Figure S3, Forest plot of odds ratio for the association of β-catenin expression in cytoplasm and/or nucleus with 5-year overall survival by subgroup analysis. Sub1: ≥50% expression rate; Sub 2: <50% expression rate. Figure S4, Forest plot of odds ratio for the association of β-catenin expression in cytoplasm and/or nucleus with 1-year recurrence-free survival by subgroup analysis. Sub1: ≥50% expression rate; Sub 2: <50% expression rate. Figure S5, Forest plot of odds ratio for the association of β-catenin expression in cytoplasm and/or nucleus with 3-year recurrence-free survival by subgroup analysis. Sub1: ≥50% expression rate; Sub 2: <50% expression rate. Figure S6, Forest plot of odds ratio for the association of β-catenin expression in cytoplasm and/or nucleus with 5-year recurrence-free survival by subgroup analysis. Sub1: ≥50% expression rate; Sub 2: <50% expression rate. Figure S7, Forest plot of odds ratio for the association of β-catenin expression in cytoplasm and/or nucleus with metastasis by subgroup analysis. Sub1: ≥50% expression rate; Sub 2: <50% expression rate. Figure S8, Forest plot of odds ratio for the association of β-catenin expression in cytoplasm and/or nucleus with vascular invasion by subgroup analysis. Sub1: ≥50% expression rate; Sub 2: <50% expression rate. Figure S9, Funnel plot to assess publication bias. A, Begg's publication bias plot showed no publication bias for studies regarding cytoplasmic and/or nuclear expression of β-catenin and 1-year overall survival (OS) in the meta-analysis. B, Begg's publication bias plot showed the presence of publication bias for studies regarding cytoplasmic and/or nuclear expression of β-catenin and 3-year OS in the meta-analysis. C, Begg's publication bias plot showed the presence of publication bias for studies regarding cytoplasmic and/or nuclear expression of β-catenin and 5-year OS in the meta-analysis. Figure S10, Funnel plot to assess publication bias. A, Begg's publication bias plot showed no publication bias for studies regarding cytoplasmic and/or nuclear expression of β-catenin and 1-year reccurrence-free survival (RFS) in the meta-analysis. B, Begg's publication bias plot showed no publication bias for studies regarding cytoplasmic and/or nuclear expression of β-catenin and 3-year RFS in the meta-analysis. C, Begg's publication bias plot showed no publication bias for studies regarding cytoplasmic and/or nuclear expression of β-catenin and 5-year RFS in the meta-analysis. Figure S11, Funnel plot to assess publication bias. Begg's publication bias plot showed no publication bias for studies regarding cytoplasmic and/or nuclear expression of β-catenin and metastasis in the meta-analysis. Figure S12, Funnel plot to assess publication bias. Begg's publication bias plot showed no publication bias for studies regarding cytoplasmic and/or nuclear expression of β-catenin and vascular invasion in the meta-analysis. Figure S13, Funnel plot to assess publication bias. Begg's publication bias plot showed the presence of publication bias for studies regarding cytoplasmic and/or nuclear expression of β-catenin and differentiation grade in the meta-analysis. Figure S14, Funnel plot to assess publication bias. Begg's publication bias plot showed no publication bias for studies regarding cytoplasmic and/or nuclear expression of β-catenin and TNM stage ((III/IV versus I/II)) in the meta-analysis. Figure S15, Funnel plot to assess publication bias. Begg's publication bias plot showed no publication bias for studies regarding cytoplasmic and/or nuclear expression of β-catenin and liver cirrhosis in the meta-analysis. Figure S16, Funnel plot to assess publication bias. Begg's publication bias plot showed the presence of publication bias for studies regarding cytoplasmic and/or nuclear expression of β-catenin and tumor size in the meta-analysis. Figure S17, Funnel plot to assess publication bias. Begg's publication bias plot showed the presence of publication bias for studies regarding cytoplasmic and/or nuclear expression of β-catenin and tumor encapsulation in the meta-analysis. Figure S18, Funnel plot to assess publication bias. Begg's publication bias plot showed no publication bias for studies regarding cytoplasmic and/or nuclear expression of β-catenin and AFP in the meta-analysis. Figure S19, Funnel plot to assess publication bias. A, publication bias plot showed no publication bias for studies regarding cytoplasmic and/or nuclear expression of β-catenin and HBV in the meta-analysis. B, Begg's publication bias plot showed no publication bias for studies regarding cytoplasmic and/or nuclear expression of β-catenin and HCV in the meta-analysis.
(RAR)
